# The Song of the Earth

**DOI:** 10.1093/function/zqac057

**Published:** 2022-11-04

**Authors:** Ole H Petersen

**Affiliations:** School of Biosciences, Sir Martin Evans Building, Cardiff University, Wales CF10 3AX, UK

Gustav Mahler’s famous valedictory song cycle “Das Lied von der Erde” (The Song of the Earth), dealing with the eternal cycle of death and (re)birth, has always been very close to my heart. Just now, only a couple of weeks after the death of Tullio Pozzan, one of *Function*’s most eminent Executive Editors, it seems particularly poignant. In the spirit of Mahler’s song cycle, this editorial is partly a short “In Memoriam” for this great scientist (*Function* will publish a more detailed obituary in due course) and partly a brief account of plans for our journal as we move on, into 2023.

Tullio Pozzan passed away on October 15, 2022, at the far too early age of 73, after a short but very rapidly developing illness. I had known Tullio for more than 40 yr and he had been a constant and crucially important friend, adviser, and constructive critic for most of my professional life. When I invited Tullio, in the autumn of 2019, to join the inaugural group of Executive Editors of *Function*, he immediately accepted. When discussing the plans for the journal in more detail—at the symposium celebrating Tullio’s 70th birthday (Figure 1) held at the University of Padua in October 2019 in the magnificent Aula Magna in Palazzo Bo, where Galileo Galilei had frequently lectured—it became clear that Tullio was genuinely enthusiastic about this new scientific journal. This was important for me, as it gave me confidence that *Function* could actually achieve something significant for the physiological community.

**Figure 1.**
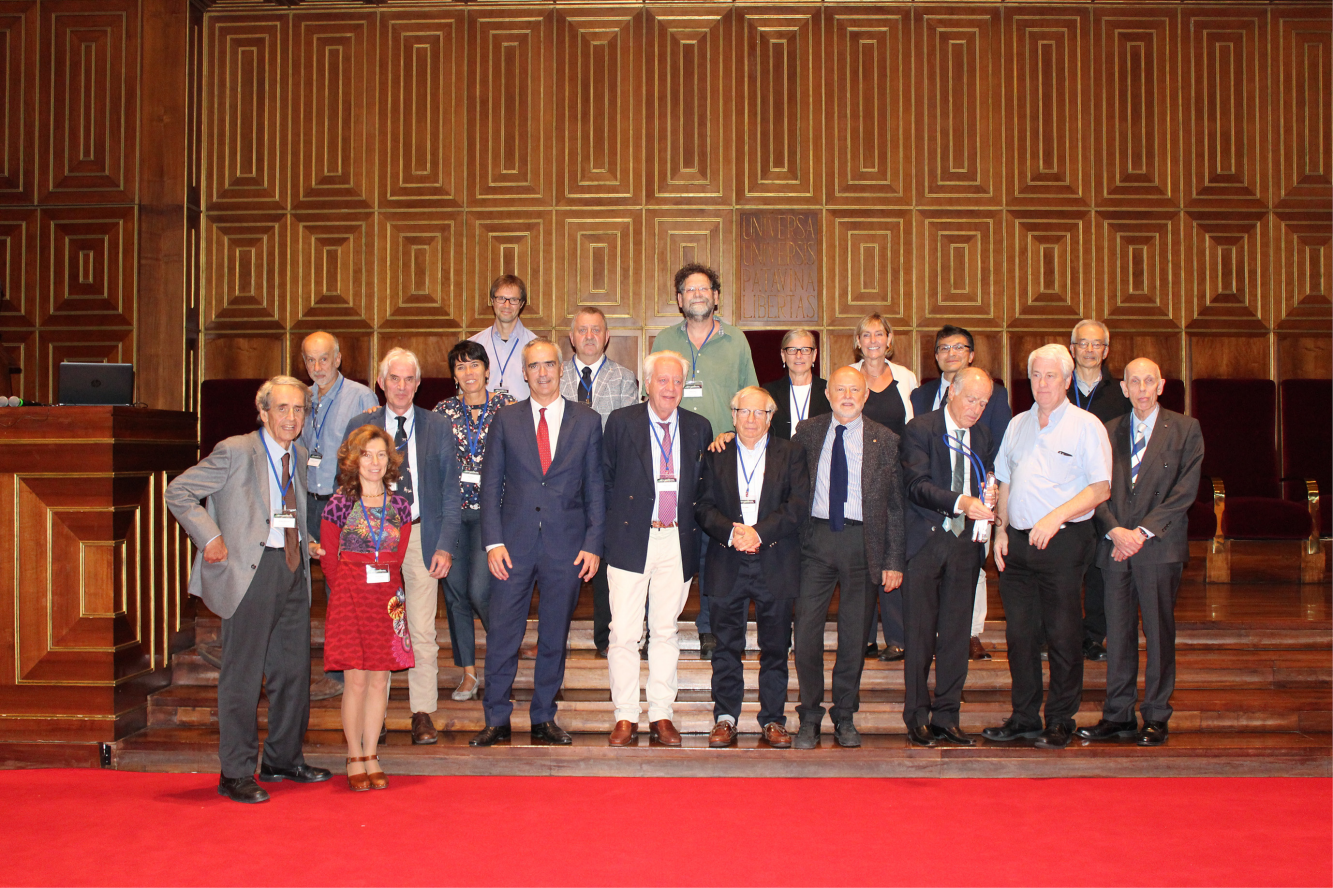
Photo of the invited speakers at the symposium celebrating Tullio Pozzan’s 70th birthday, held at the University of Padua’s Aula Magna in Palazzo Bo on October 12, 2019. Tullio Pozzan is seen in the middle of the front row (blue jacket and white trousers) between Rosario Rizzuto (red tie) to the left and Tim Rink (wearing glasses) to the right. Several editors and editorial board members of *Function* are also seen in the picture. In the front row: Ole Petersen, first from the right; and Andrew Thomas, second from the right. In the back row: Anant Parekh, second from the right; Aldebaran Hofer, third from the right; and Manuela Zaccolo, fourth from the right.

Tullio was universally recognized as one of the most eminent biologists of his generation. He received much well-deserved recognition, including election to numerous important academies, for example, the Academia dei Lincei (2001), the US National Academy of Sciences (2006), and the Royal Society (2018). For almost the whole of his professional life, he worked at the University of Padua in Italy, except for a period (1978–1981) as a Long Term EMBO Fellow at the University of Cambridge.

During his Cambridge period, he was a part of the trio [with Roger Tsien (Chemistry Nobel Prize 2008) and Tim Rink] that developed the revolutionary technique for measurements of Ca^2+^ in living cells with fluorescent indicators that could be trapped intracellularly.^1,2^ This new technique had a colossal influence on the Ca^2+^ signaling field and rapidly became the dominant technique for assessing changes in the cytosolic Ca^2+^ concentration, a key parameter in the control of almost all cellular activities.

Tullio’s most important work was carried out at the University of Padua, in close collaboration with his younger co-worker Rosario Rizzuto. They developed the first genetically encoded probes for Ca^2+^ measurements in selected subcellular localizations. With the help of this new technique, they published a landmark paper in *Science*, demonstrating for the first time that microdomains with a high Ca^2+^ concentration close to IP_3_-sensitive channels in the endoplasmic reticulum (ER) membrane are sensed by neighboring mitochondria.^3^ This paper initiated a revolution in our understanding of the role of mitochondria in cellular Ca^2+^ homeostasis. It also solved a key problem in cellular physiology, namely, how increased cell activity—elicited by cytosolic Ca^2+^ signals—was linked to the required increase in mitochondrial ATP production.

The 1993 *Science* paper^3^ started a new era of cellular signaling work with a focus on subcellular microdomains, also reflected in other work published that same year.^4^ In 1998, a further *Science* paper by Tullio and his collaborators^5^ provided impressive high-resolution evidence for linkage between local Ca^2+^ signals, generated by release from the ER, and mitochondrial Ca^2+^ uptake. This Ca^2+^ uptake stimulates the Krebs cycle, thereby generating ATP that is needed to power cellular activity.

Tullio continued to generate useful and important tools for cellular physiologists, also focussing on another critical intracellular messenger molecule, namely, cyclic adenosine monophosphate (cyclic AMP). He generated the first genetically encoded fluorescent probe for this messenger and established the concept of cyclic AMP microdomains.^6^

More recently, Tullio published an important paper in *Function*, describing a new transgenic mouse line for imaging mitochondrial Ca^2+^ signals.^7^ Anant Parekh, in his accompanying Perspective, wrote: “The erudite, innovative and timely advance takes us to the next level, the ability to study mitochondrial Ca^2+^ signaling in vivo. This landmark contribution will no doubt lead to new vistas in Ca^2+^ signaling in health and disease.” ^8^

Tullio’s death is a heavy loss for the cellular signaling community and for *Function*. As we mourn Tullio, we nevertheless have to continue our work and we need to plan for the future of *Function*. In this context, I am very pleased to report that Annette Dolphin FRS, Professor of Pharmacology at University College London, has accepted the invitation to join the group of Executive Editors from January 1, 2023. She has been a member of the editorial board of *Function* from the beginning and also been a consistent and important contributor. Annette’s innovative work on voltage-gated Ca^2+^ channels has led the field for many years.^9^

One of the most important problems facing every new journal is the long time it takes before its content is listed in the most important databases, even when the journal has a distinguished editorial board and is published by major well-known organizations. Happily, this period has now come to an end for our journal. *Function* has already for some time been included in PubMed Central and Web of Knowledge (Clarivate). More recently, Scopus has also started to list our content and this is now visible.

From the very start of *Function*, it was planned to combine publication with in-person scientific conferences. However, the COVID pandemic prevented this from happening. Now that the world is opening up, we are planning a *Function* symposium early in 2023. Following the remarkable interest in the editorial published in *Function*’s previous issue (“Are scientists sufficiently ambitious?”),^10^ the symposium will be ambitious, aiming to capture, assess, and discuss the most important progress in the broad areas of physiology and pathophysiology. We do not want to drown in data, but rather aim to gain real and useful knowledge.^10^ We have a stellar list of speakers and much time will be devoted to interactive discussions. The symposium will take place in the heart of Europe, at the German National Academy of Sciences Leopoldina in Halle on March 7 and 8, 2023. The conference is co-sponsored by the American Physiological Society and the Leopoldina Academy and organized in co-operation with Academia Europaea’s Cardiff University Knowledge Hub. Registration will soon open and all details will be available at https://aecardiffknowledgehub.wales/2022/10/21/physiology-pathophysiology-2023-symposium/

The symposium will be a unique opportunity to gain a high-level overview of where Physiology is right now and to learn from world leaders in the field where we are heading.

## Funding

None declared.
